# Supplementary material for: From Random Perturbation to Precise Targeting: A Comprehensive Review of Methods for Studying Gene Function in Monascus Species
Source: J Fungi (Basel). 2024 Dec 23;10(12):892. doi: 10.3390/jof10120892 (PMC11678829; doi:10.3390/jof10120892)
Supplement: Supplementary file 1 [file jof-10-00892-s001.zip › supplementary table.pdf]

Table S1 Strategies for growth, development, and metabolic regulation in *Monascus* spp..

Genomic perturbation	Strategy	Item	Details	Metabolite regulation	Refs
No	Optimization of cultural conditions	Carbon/Nitrogen sources	Rice straw hydrolysate	Pigment yield was reached to 20.86U/mL, increased by 72.68%	[1]
			Glycerol	Pigment yield was reached to 2.7 U/mL, increased by 200%	[2]
			Glutamic	Pigment production increased by 2.82%	[3]
			Soybean	Production of GABA and free amino acids were increased by 149.21% and 234.06%	[3]
			Chicken feather peptone	Pigments values were reached to 2.315, 1.992 and 2.217 UA/10mL, respectively	[4]
		pH	Low pH (pH 2.5 and 4.0)	Intracellular pigments dominated by orange pigments	[5]
		Temperature	Variable temperature fermentation	MK yield was reached to 236.8 mg/L, increased by 393.33%	[6]
Yes	Random perturbation	Physical mutagenesis	Treatment with UV irradiation for 7 min	30%, 18% and 27% increases in yields of MK, GABA and MonAzPs	[7]
			ARTP	One mutant showing an increase of 66.7% in orange pigment was obtained	[8]
		Chemical mutagenesis	Treatment of 2 mg/ml EMS	Yields of MK and MonAzPs in M. sanguineus were increased by 20.83% and 163.34%	[7]
		Protoplast fusion	Intergeneric hybridization	2.5 times higher MK yields	[9]
		PMT	REMI/aurintricarboxylic acid (ATA) treatment	Red-pigment production of transformant was increased by 600%	[10]
		Physical transformation	BT	Two transformants did not produce ascospores	[11]
			EP	Six mutants with ability of pigment synthesis were obtained using UV-induced albino mutant as parent	[12]
		ATMT	T-DNA mutation library	Four mutants with increased pigment yields were obtained from 5132 transformants, among which, pigment yield was increased by 120.85%	[13]
	Precise targeted manipulation		Site-specific gene editing achieved by HDR	Improved strains of reduced CIT content and increased pigment and MK yields have been constructed for enhancing HDR by sequence analysis and bioinformatics methods	[14]
		CRISPR/Cas system	Site-specific gene editing achieved by HDR	Knocking out 15-kb CIT BGC, resulting 2-5% increases in MonAzPs production	[15]
			Site-specific gene editing achieved by HDR	A Cas9-mediated mutant formed 18.5 times more red pigments than wild-type strain.	[16]
			Site-specific gene editing achieved by HDR	MK yield was increased by 52.6%	[17]
		Base editing	Site-specific gene editing achieved by HDR	Mutant with a 10-fold increase in MK production was obtained	[18]

References

1. Liu, J.; Luo, Y.; Guo, T.; Tang, C.; Chai, X.; Zhao, W.; Bai, J.; Lin, Q. Cost-effective pigment production by Monascus purpureus using rice straw hydrolysate as substrate in submerged fermentation. Journal of bioscience and bioengineering 2020, 129, 229-236
2. Shi, J.; Zhao, W.; Lu, J.; Wang, W.; Feng, Y. Insight into Monascus pigments production promoted by glycerol based on physiological and transcriptome analyses. Process Biochemistry 2020, 102.
3. Zhang, X., Wang, J., Chen, M., Wang, C.. Effect of nitrogen sources on production and photostability of Monascus pigments in liquid permentation. IERI Procedia 2013, 5: 344-350.
4. Orak T, Caglar O, Ortucu S, Ozkan H, Taskin M. Chicken feather peptone: A new alternative nitrogen source for pigment production by Monascus purpureus. J Biotechnol. 2018,10;271:56-62.
5. Shi K, Song D, Chen G, Pistolozzi M, Wu Z, Quan L. Controlling composition and color characteristics of Monascus pigments by pH and nitrogen sources in submerged fermentation. J Biosci Bioeng. 2015, 120(2):145-54.
6. Huang, C., Shen, S., Chen, W., Chen, C.. The effects of mutation and temperature variation on monacolin K production by monascus sp. and relative statistical parameter analysis of monacolin K production. Phytochemistry Letters.2019,32:143-150
7. Dikshit, R.,Tallapragada, P.. Development and screening of mutants from Monascus sanguineus for secondary metabolites production. Beni-Suef University Journal of Basic and Applied Sciences.2018,7(2),235-240
8.Zhang C, Sun Q, Yang L, Ablimit A, Dong H, Wang H, Wang C, Wang C. Mutation breeding of Monascus to produce a high yield of orange pigment and low citrinin content using the ARTP method. J Fungi (Basel). 2024,10(8):553.
9. Sinha, A., Sarkar, K.. Enhancement of Monacolin K production by intergeneric hybridization between Monascus purpureus (Arg-) and Monascus ruber (Thi-, Met-) auxotrophs. Journal of Pure and Applied Microbiology.2023, 17(3):1458-1470
10. Chen YP, Chen IC, Hwang IE, Yuan GF, Liaw LL, Tseng CP. Selection of an effective red-pigment producing Monascus pilosus by efficient transformation with aurintricarboxylic acid. Biosci Biotechnol Biochem. 2008,72(11):3021-3024.
11. Lakrod, K., Chaisrisook, C.,Skinner, D.Z. Transformation of Monascus purpureus to hygromycin B resistance with cosmid pMOcosX reduces fertility. Electronic Journal of Biotechnology, 2003,6, 143-147.
12. Lakrod K, Chaisrisook C, Skinner DZ. Expression of pigmentation genes following electroporation of albino Monascus purpureus. J Ind Microbiol Biotechnol. 2003, 30(6):369-374.
13. Yanchun, S.; Ruyi, W.; Yuedi, D.; Fusheng, C.; Bijun, X. Construction of T-DNA insertional library of Monascus mediated by Agrobacterium tumefaciens and characteristic analysis of the color mutants. Mycosystema (Chinese) 2006, 25, 247-255
14. Shao Y, Lei M, Mao Z, Zhou Y, Chen F. Insights into Monascus biology at the genetic level. Appl Microbiol Biotechnol. 2014 ,98(9):3911-22.
15. Liu W, An C, Shu X, Meng X, Yao Y, Zhang J, Chen F, Xiang H, Yang S, Gao X, Gao SS. A dual-plasmid CRISPR/Cas system for mycotoxin elimination in polykaryotic industrial fungi. ACS Synth Biol. 2020, 21;9(8):2087-2095.
16. Ree Yoon H, Han S, Chul Shin S, Cheong Yeom S, Jin Kim H. Improved natural food colorant production in the filamentous fungus Monascus ruber using CRISPR-based engineering. Food Res Int. 2023,167:112651.
17. Gong Y, Li S, Liu Q, Chen F, Shao Y. CRISPR/Cas9 system is a suitable gene targeting editing tool to filamentous fungus Monascus pilosus. Appl Microbiol Biotechnol. 2024,108(1):154
18. Duan Y, Tan Y, Chen X, Pei X, Li M. Modular and Flexible Molecular Device for Simultaneous Cytosine and Adenine Base Editing at Random Genomic Loci in Filamentous Fungi. ACS Synth Biol. 2023,12(7):2147-2156.
