# Supplementary figures and images for: From Random Perturbation to Precise Targeting: A Comprehensive Review of Methods for Studying Gene Function in Monascus Species
Source: J Fungi (Basel). 2024 Dec 23;10(12):892. doi: 10.3390/jof10120892 (PMC11678829; doi:10.3390/jof10120892)

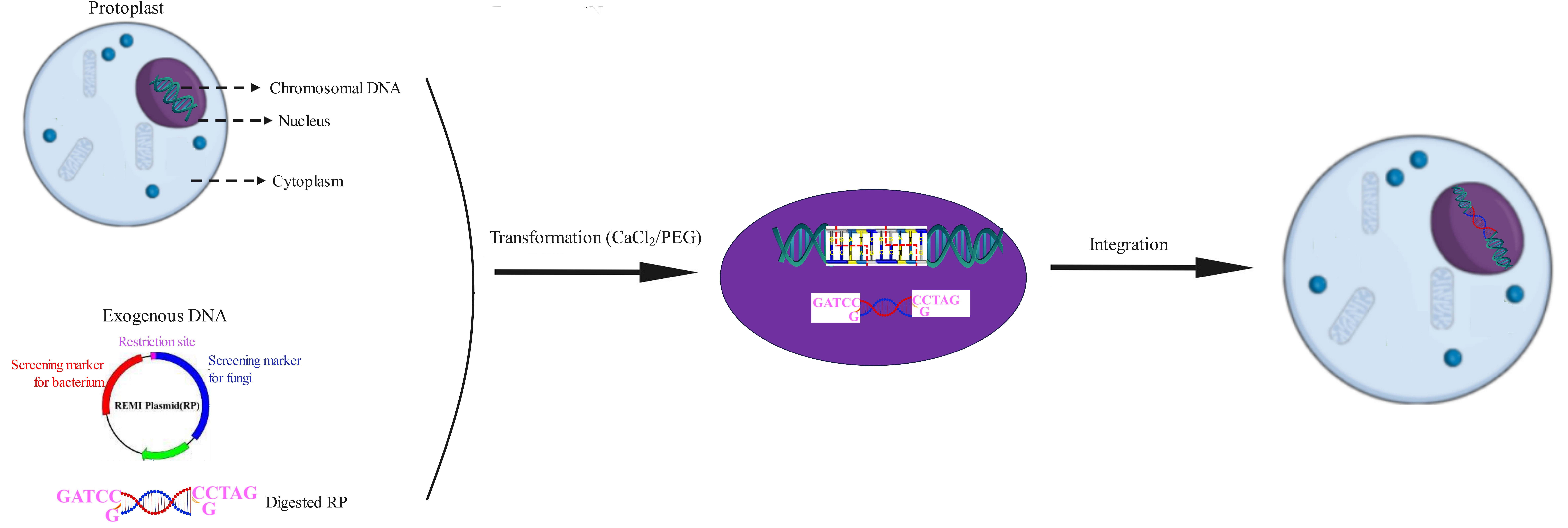

Supplement: Supplementary file 1 [file jof-10-00892-s001.zip › Fig. S1.jpg]

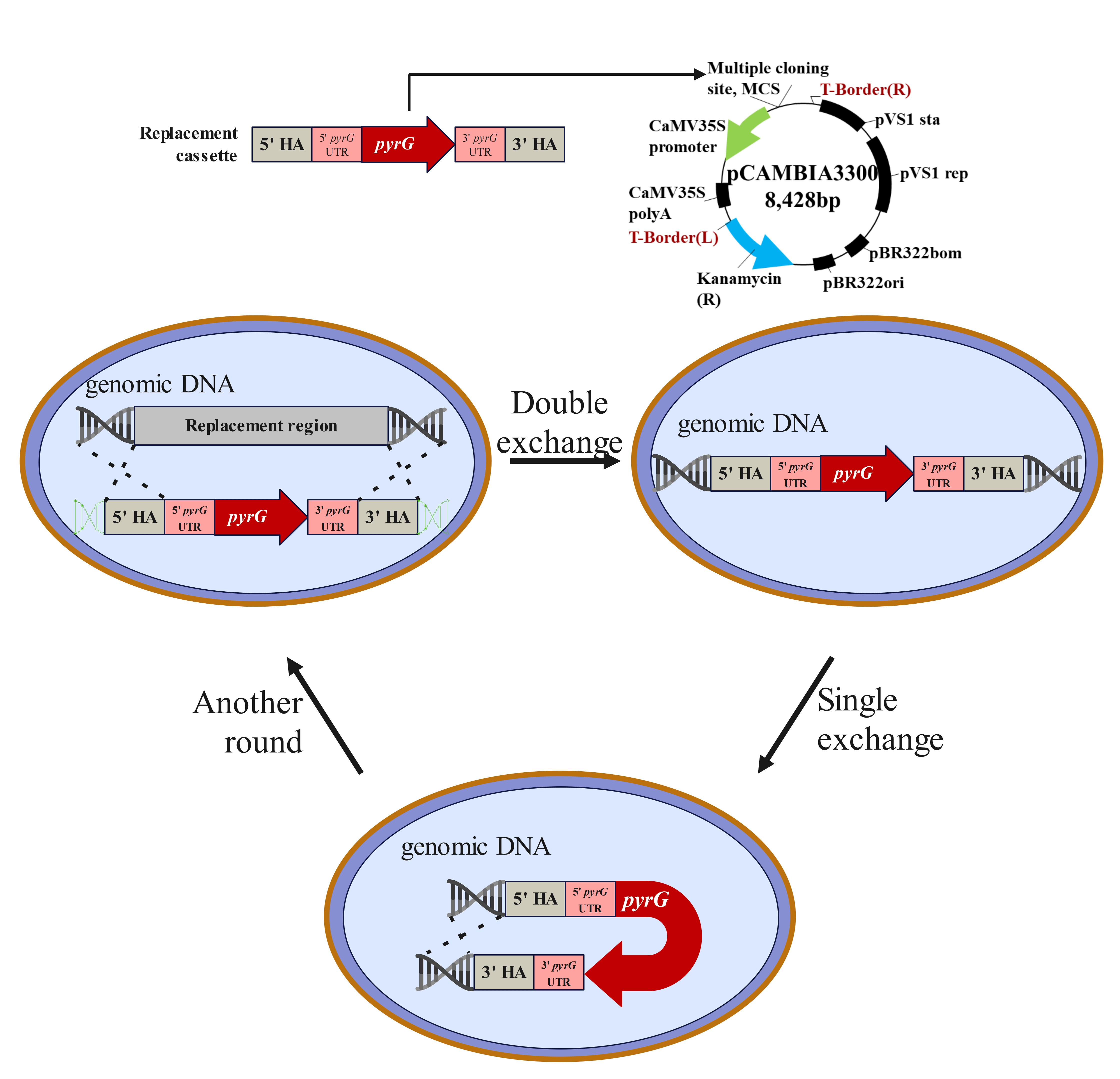

Supplement: Supplementary file 1 [file jof-10-00892-s001.zip › Fig. S2.jpg]

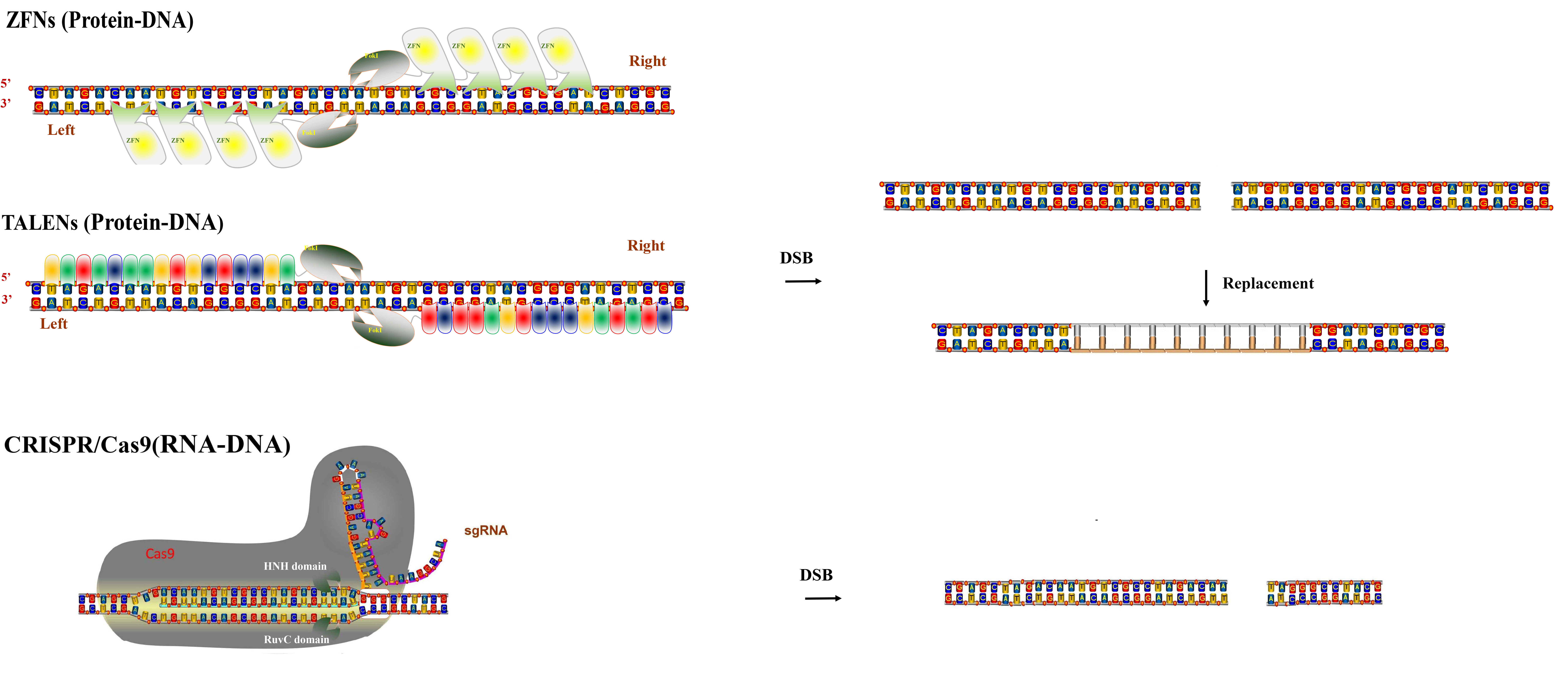

Supplement: Supplementary file 1 [file jof-10-00892-s001.zip › Fig. S3.jpg]
